# Epigenetic mechanisms differentially regulate blood pressure and renal dysfunction in male and female *Npr1* haplotype mice

**DOI:** 10.1096/fj.202400714R

**Published:** 2024-08-07

**Authors:** Prerna Kumar, Kandasamy Neelamegam, Chandramohan Ramasamy, Ramachandran Samivel, Huijing Xia, Daniel R. Kapusta, Kailash N. Pandey

**Affiliations:** ^1^ Department of Physiology School of Medicine, Tulane University Health Sciences Center New Orleans Louisiana USA; ^2^ Department of Pharmacology Louisiana State University Health Sciences Center New Orleans Louisiana USA

**Keywords:** epigenetics, hypertension, miR‐133a, mocetinostat, natriuretic peptide receptor‐A, NF‐κB, renal dysfunction

## Abstract

We determined the epigenetic mechanisms regulating mean arterial pressure (MAP) and renal dysfunction in guanylyl cyclase/natriuretic peptide receptor‐A (GC‐A/NPRA) gene‐targeted mice. The *Npr1* (encoding NPRA) gene‐targeted mice were treated with class 1 specific histone deacetylase inhibitor (HDACi) mocetinostat (MGCD) to determine the epigenetic changes in a sex‐specific manner. Adult male and female *Npr1* haplotype (1‐copy; *Npr1* ^*+/−*^), wild‐type (2‐copy; *Npr1* ^*+/+*^), and gene‐duplicated heterozygous (3‐copy; *Npr1* ^*++/+*^) mice were intraperitoneally injected with MGCD (2 mg/kg) for 14 days. BP, renal function, histopathology, and epigenetic changes were measured. One‐copy male mice showed significantly increased MAP, renal dysfunction, and fibrosis than 2‐copy and 3‐copy mice. Furthermore, HDAC1/2, collagen1alpha‐2 (Col1α‐2), and alpha smooth muscle actin (α‐SMA) were significantly increased in 1‐copy mice compared with 2‐copy controls. The expression of antifibrotic microRNA‐133a was attenuated in 1‐copy mice but to a greater extent in males than females. NF‐κB was localized at significantly lower levels in cytoplasm than in the nucleus with stronger DNA binding activity in 1‐copy mice. MGCD significantly lowered BP, improved creatinine clearance, and repaired renal histopathology. The inhibition of class I HDACs led to a sex‐dependent distinctive stimulation of acetylated positive histone marks and inhibition of methylated repressive histone marks in *Npr1* 1‐copy mice; however, it epigenetically lowered MAP, repaired renal fibrosis, and proteinuria and suppressed NF‐kB differentially in males versus females. Our results suggest a role for epigenetic targets affecting hypertension and renal dysfunction in a sex‐specific manner.

## Graphical abstract

Signaling of natriuretic peptides and their cognate natriuretic peptide receptor‐A (NPRA) lowers blood pressure and fluid volume. *Npr1* haplotype (1‐copy) male mice exhibited significantly higher mean arterial pressure, renal fibrosis, and histone‐specific epigenetic changes as compared with 1‐copy female mice. Mocetinostat (MGCD), a class I HDAC inhibitor, enhanced the expression of *Npr1* and anti‐fibrotic miR‐133a and suppressed the HDAC activity, renal fibrotic markers, and NF‐κB signaling in 1‐copy mice with greater efficacy in males than females.

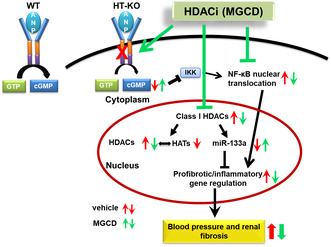

**Abbreviations**
ANP: atrial natriuretic peptide
ATRA: all‐trans retinoic acid
BNP: brain natriuretic peptide
CKD: chronic kidney disease
COL1α‐2: collagen1 alpha‐2
GC‐A/NPRA: guanylyl cyclase/natriuretic peptide receptor‐A
H3: histone 3
HAT: histone acetyltransferase
HDAC: histone deacetylase
HDACi: HDAC inhibitor
MAP: mean arterial pressure
me: methylation
MGCD: mocetinostat
miR: microRNA
MME: mesangial matrix expansion
SBP: systolic blood pressure
α‐SMA: alpha‐smooth muscle actin

## 1 INTRODUCTION

Atrial and brain natriuretic peptides (ANP and BNP) activate guanylyl cyclase/natriuretic peptide receptor‐A (GC‐A/NPRA), which produces the intracellular second messenger cyclic GMP in response to hormone binding.^1^, ^2^, ^3^, ^4^ The ANP/NPRA signaling enhances the excretion of Na^+^ and water, thereby prevents hypertension and cardiac injury and remodeling.^5^, ^6^, ^7^ The complete systemic absence of *Npr1* is associated with congestive heart failure (CHF) and sudden death after 6 months of age.^8^, ^9^, ^10^ Genetic studies have demonstrated the association of polymorphisms and allelic variants of ANP (*Nppa*), BNP (*Nppb*), and GC‐A/NPRA (*Npr1*) with a family history of hypertension, increased left ventricular mass, and cardiovascular diseases (CVD) in humans.^11^, ^12^, ^13^, ^14^

Epigenetic mechanisms of histone modifications (acetylation and methylation) modulate gene expression and function in pathophysiology of the renal disorders and CVD.^15^, ^16^, ^17^, ^18^, ^19^ The balance between the acetylated and deacetylated histone marks is mediated by two different sets of enzymes: histone acetyltransferases (HATs), which promote histone lysine acetylation and activate gene transcription, and histone deacetylases (HDACs), which catalyze deacetylation of histones and repress gene transcription. There are four classes of mammalian HDACs. The members of class I HDACs (HDAC1, 2, 3, and 8) are widely expressed and implicated in the renal and CVD.^16^, ^20^, ^21^ The evidence suggests that the inhibition of HDACs may also epigenetically regulate expression of microRNAs (miRs).^17^, ^22^ Most miRs are small noncoding RNA, which consists of 18‐22 nucleotides that comprise a large family of posttranscriptional regulators of gene expression.^23^ The miR‐133 family of genes appear to be dysregulated in hypertension and CVD^18^, ^24^; however, the underlying interactive roles of specific HDAC isoforms, modified histone marks, miR‐133, and genetic and epigenetic signaling mechanism(s) in the control of blood pressure (BP), renal injury, and disease pathology remain unclear.

Sex‐specific differences of epigenetic mechanisms regulating BP and renal disorders are poorly understood. Sex is increasingly recognized as a biological modulator of renal function; however, the existence of sex‐related variations in the maintenance of the chronic arterial pressure, hypertension, and renal injury and dysfunction is not well studied. Understanding the mechanisms underlying sex‐specific differences in the epigenetic regulation of high BP and renal dysfunction involving *Npr1* may reveal new direction to study hypertension and related diseases.^25^, ^26^, ^27^, ^28^ The interest and awareness of the differential effects of drugs in hypertensive male versus female patients has produced heightened interest in research aimed at elucidating sex responses. In the present study, we aimed to determine the epigenetic regulation of high BP and renal dysfunction in *Npr1* gene‐targeted mice in a sex‐specific manner. Haplotype/heterozygous mice (having only one *Npr1* allele) exhibit a loss‐of‐function mutation, which could be comparable to the *Npr1* gene polymorphisms found in the human population.^29^, ^30^, ^31^ The recent studies have indicated that genetic and epigenetic mechanisms play the pivotal roles in the regulation of BP and kidney injury and dysfunction.^32^ In the current work, we used global *Npr1* gene‐targeted male and female mice to determine the effect of class I selective HDAC inhibitor (HDACi), mocetinostat (MGCD0103; MGCD), to identify the role of class I HDACs in sex‐specific differential regulation of hypertension and renal dysfunction and injury. Further, we examined whether HDAC1 and 2 differentially regulate interrelated downstream targets, including miR‐133a, fibrotic markers, such as α‐smooth muscle actin (α‐SMA) and collagen1α‐2 (COL1α‐2), and transcription factor, nuclear factor‐kappa B (NF‐κB) in male and female *Npr1* mice, and affect sex‐specific epigenetic mechanisms, regulating high BP and renal dysfunction in mutant animals.

## 2 MATERIALS AND METHODS

### 2.1 Animals and treatments

*Npr1* gene‐disrupted and gene‐duplicated mice were produced by homologous recombination in embryonic stem cells as previously described.^8^, ^33^, ^34^ The mice were bred and maintained at the Tulane University School of Medicine animal care facility and managed under the protocols approved by the Institutional Animal Care and Use Committee. The mouse colonies were housed under 12‐h light and 12‐h dark cycles at 25°C and fed regular chow (Purina Laboratory, Framingham, MA, USA) and tap water ad libitum. All animals were littermate progeny of the C57/BL6 genetic background and were designated as *Npr1* gene‐knockout heterozygous/haplotype (HT) (1‐copy; *Npr1* ^*+/−*^), wild‐type (WT) control (2‐copy; *Npr1* ^*+/+*^), and gene‐duplicated HT (3‐copy; *Npr1* ^*++/+*^) mice. Stock solution (10 μg/μl) of mocetinostat (MGCD0103; MGCD) was prepared in dimethyl sulfoxide (DMSO) and frozen at −80°C. On the day of injection, MGCD stock solution was thawed and diluted with olive oil to the appropriate concentrations. Adult (16–18 weeks) *Npr1* male and female 1‐copy, 2‐copy, and 3‐copy mice were intraperitoneally injected with vehicle (DMSO and olive oil; control group; *n* = 12) and MGCD (2 mg/kg; treated group; *n* = 12) for 2‐week. The mean arterial pressure (MAP) was determined by radiotelemetry procedure.

### 2.2 Implantation of radiotelemetry probe and BP measurement

Blood pressure (BP) was recorded in chronically catheterized mice using radiotelemetry method (Data Sciences International, New Brighton, MN, USA) as previously described.^35^, ^36^ The transmitter was turned on magnetically for 24 h and soaked in sterile saline solution for at least 10 min prior to surgery. Mice were anesthetized with isoflurane 3%–5% induction and 0.5%–1% maintenance. A 50:50 solution of bupivacaine and lidocaine was administered subcutaneously at the surgical site prior to creation of incision for analgesia. The left carotid artery was exposed, isolated, and temporarily occluded. The artery was cannulated with catheter portion of the transmitter (model TA11PH‐10, Data Sciences International) and the main body of the device was secured inside a skin pocket fashioned on the right flank of the animal. Placement of catheter was verified by the quality of the audio output signal on FM radio. The skin was sutured, and the animals were closely observed and kept warm until recovery. The analgesic buprenorphine (BuprenexTM: 0.05–0.1 mg/kg body weight) was given subcutaneously to alleviate postoperative pain and distress and mice were monitored. Animals remained undisturbed in their home cages on top of a telemetry receiving plate for 7 days before the initiation of studies. The data was recorded by a computer program (Dataquest, St. Paul, MN, USA) for continuous monitoring and collection of data at predetermined intervals. BP waveforms were sampled at a rate of 1000 Hz for 10 s every 10 min. MAP was averaged weekly during light (6:00 AM to 6:00 PM) and dark (6:00 PM to 6:00 AM) periods to record the BP throughout the 2 weeks of the experiment.

### 2.3 Blood pressure measurements by tail‐cuff method

Systolic blood pressure (SBP) of mice was measured with a noninvasive computerized tail‐cuff method using Visitech BP2000 (Visitech Systems Inc., Apex, NC, USA) as previously described.^37^ All mice were trained for BP measurement (acclimatization) for 5 days and the actual BP was calculated as the average of 5 sessions from day 13 to 15 of the treatment.

### 2.4 Blood and tissue collection

Mice were anesthetized using CO_2_ and blood was collected by cardiac puncture in prechilled tubes containing EDTA. Plasma was separated by centrifugation at 3000 rpm for 10 min and stored at −80°C. Mice were euthanized by administration of a high concentration of CO_2_ and kidneys were isolated, dissected, frozen in liquid nitrogen, and stored at −80°C. One half from each kidney tissue sample was stored overnight in 10% buffered formalin and processed for histological studies.

### 2.5 Histopathological analysis

Kidney tissues from vehicle‐ and drug‐treated mice groups were fixed in 10% buffered‐ formalin solution. Paraffin‐embedded tissue sections (5 μm) were stained with hematoxylin and eosin (H&E) to study renal morphology as previously described.^38^ Kidney sections were stained with Picrosirius red to observe the presence of collagen fiber accumulation (red color) as a marker of renal fibrosis. Photomicrographs of renal tissue sections were obtained using Olympus BX51 microscope in a blinded manner. Percentage of the MME, interstitial nephritis, and fibrosis relative to the total kidney area was determined by calculations made in 20 randomly selected microscopic fields in five sections per kidney using ImagePro Plus image analysis software (Media Cybernetics, Silver Spring, MD, USA).

### 2.6 Real‐time RT‐PCR assay

Pooled kidney tissue (30 mg) from 6 mice was used to extract total RNA using RNeasy Plus mini‐kit (Qiagen, Valencia, CA, USA). First‐strand cDNA was synthesized from 1 μg of total RNA in a final volume 20 μL using Transcriptor First Strand cDNA synthesis kit (Roche Diagnostics, Indianapolis, IN, USA). Primers for *Npr1* and β‐actin were purchased from Qiagen. Real‐time‐PCR amplification was performed using the RT2 Real‐Time SYBR Green/ROX PCR Master Mix (Qiagen, Germantown, MD, USA) in Mx3000P real‐time PCR system and data were analyzed with MxPro software (Stratagene, La Jolla, CA, USA). The reaction mixture without template cDNA was used as a negative control. The *Npr1* expression values were normalized to β‐actin and relative expression of the *Npr1* gene was determined using the comparative cycle threshold (Ct) method as detailed earlier.^39^ Male and female samples were run on the same plate using similar PCR cycles.

### 2.7 Cytosolic and nuclear extract preparation

Cytosolic and nuclear extracts were prepared from frozen kidney tissues as described previously.^38^, ^40^ Briefly, pooled kidney tissues were homogenized in an ice‐cold 10 mM Tris‐HCl buffer (pH 8.0) containing, 3 mM CaCl_2_, 0.32 M sucrose, 2 mM magnesium acetate (MgOAc), 0.1 mM EDTA, 0.5% nonidet P‐40 (NP‐40), 1 mM dithiothreitol (DTT), 0.5 mM phenylmethylsulfonyl fluoride (PMSF), 1 mM orthovanadate and 30 mM sodium fluoride (NaF) and 10 μg/mL each of leupeptin and aprotinin. The homogenate was centrifuged at 8000 ***g*** at 4°C for 20 min and the supernatant was stored as cytosolic fraction. The pellet was washed 3× with wash buffer (same as above except NP‐40) by resuspending with a 20‐gauge needle and centrifugation at 6000 ***g***. After the final wash, the pellet was resuspended in a low‐salt buffer (20 mM HEPES, pH 7.9, 1.5 mM MgCl_2_, 20 mM KCl, 0.2 mM EDTA, 25% glycerol, 0.5 mM DTT, and 0.5 mM PMSF), incubated on ice for 5 min, and mixed with an equal volume of high‐salt buffer containing 20 mM HEPES (pH 7.9), 800 mM KCl, 1.5 mM MgCl_2_, 0.2 mM EDTA, 25% glycerol, 1% NP‐40, 0.5 mM DTT, 0.5 mM PMSF, 1 mM orthovanadate, 30 mM NaF, and 10 μg/mL of leupeptin and aprotinin. The mixture was incubated on ice for 30 min and centrifuged at 14 000 ***g*** for 20 min. The supernatant was separated and stored as a nuclear fraction at −80°C. The protein concentration of the extracts was measured by Bradford detection kit (Bio‐Rad, Hercules, CA, USA) using BSA as a standard.

### 2.8 Western blot analysis

Cytoplasmic fraction (40–60 μg), nuclear extracts (30–40 μg), or histones (8–10 μg) were mixed with sample loading buffer and separated by using 10% sodium dodecyl sulfate‐polyacrylamide gel electrophoresis (SDS‐PAGE) essentially as described earlier.^41^ Proteins were electrotransferred onto a polyvinylidene difluoride (PVDF) membrane and blocked with 1× Tris‐Buffered Saline‐Tween 20 (TBST; 25 mM Tris, 500 mM NaCl, and 0.05% Tween 20, pH 7.5) containing 5% fat‐free milk for 1 h. Membrane was incubated overnight at 4°C in TBST containing 5% fat‐free milk with primary antibodies (1:250–1:1000 dilution) and treated with corresponding secondary anti‐rabbit, anti‐mouse, or anti‐chicken horseradish peroxidase‐conjugated antibodies (1:5000 dilutions). Protein bands were visualized by enhanced chemiluminescence plus detection system with Alpha Innotech phosphoimager from ProteinSimple (Santa Clara, CA, USA). The antibodies used in Western blot were: NPRA (135 kDa; 15‐288‐22 960, 1:500; custom prepared from Genway Biotech Inc., San Diego, CA, USA), HDAC1 (62 kDa; 2062; 1:1000; Cell Signaling Technology (CST), Danvers, MA, USA), HDAC2 (59 kDa; 2540; 1:1000; CST, Danvers, MA, USA); HDAC3 (49 kDa; 2632; 1:1000; CST, Danvers, MA, USA); H3‐K9ac (17 kDa; 9649, 1:1000; CST, Danvers, MA, USA); H3‐K18ac (17 kDa; 13 998; 1:1000; CST, Danvers, MA, USA); H3‐K27ac (17 kDa; 8173; 1:1000; CST, Danvers, MA, USA); H3‐K4me3 (17 kDa; 39 159; 1:1000; Active Motif, Carlsbad, CA, USA); H3 (15 kDa; 4499; 1:1000; CST, Danvers, MA, USA); Alpha‐SMA (42 kDa; A‐7607; 1:1000; Sigma Aldrich St. Louis, MO, USA); Col1 A2 (70 kDa; sc‐376 350; 1:250; Santa Cruz Biotechnology (SCBT), Santa Cruz, CA, USA); IκB‐α (36 kDa; sc 371; 1:250; SCBT, Santa Cruz, CA, USA); p‐IκB‐α (41 kDa; sc 8404; 1:250; SCBT, Santa Cruz, CA, USA); NF‐κB (p65) (65 kDa; sc 372; 1:250; SCBT, Santa Cruz, CA, USA); β‐actin (42 kDa; sc 47 778 HRP; 1:5000; SCBT, Santa Cruz, CA, USA); TBP (36 kDa; sc‐271146; 1:250; SCBT, Santa Cruz, CA, USA); HRP‐conjugated anti‐mouse IgG (sc‐516102; 1:5000; SCBT, Santa Cruz, CA, USA); HRP‐conjugated anti‐rabbit IgG (7074; 1:5000; CST, Danvers, MA, USA).

### 2.9 Histone purification

Total histone was extracted from frozen kidney tissues using a total histone extraction kit from Epigentek (Farmingdale, NY, USA) according to the manufacturer's protocol as previously reported.^41^ Briefly, kidney tissues were weighed and homogenized in 1 × pre‐lysis buffer, transferred to a 2 mL tube, and centrifuged at 10 000 rpm for 1 min at 4°C. The supernatant was removed, and the tissue pellet was resuspended in 3 volumes of lysis buffer, incubated on ice for 30 min, and centrifuged at 12 000 ***g*** for 5 min at 4°C. Balance‐DTT buffer (0.3 volumes) was added to the supernatant and stored at −80°C. The protein concentration of the eluted histone was estimated using a Bradford protein detection kit.

### 2.10 HDAC and HAT activity assay

Total HDAC and HAT enzyme activities were measured in kidney nuclear extracts using colorimetric Epigenase HDAC activity/inhibition assay kit and EpiQuik HAT activity/inhibition assay kit, respectively, from Epigentek (Farmingdale, NY, USA). The HDAC activity was calculated by directly detecting the HDAC‐converted deacetylated product, which was directly proportionate to the HDAC activity. For the HAT enzyme activity assay, the amount of acetylated histone substrate was quantified as previously described.^38^ Absorbance was read at 450 nm and results were calculated using a standard curve following the manufacturer's instructions and expressed as ng/min/mg protein. Male and female samples were analyzed on the same ELISA plate.

### 2.11 Quantification of global H3 lysine methylation

Quantification of renal levels of H3 methylation at specific lysine residues in nuclear extracts was performed using EpiQuik global H3‐K9me and H3‐K27me colorimetric methylation assay kit from Epigentek following the manufacturer's protocol. In the assay, methylated histones were captured with specific antibody and detected with a labeled detection antibody, followed by an HRP‐conjugated secondary antibody‐color development system. Absorbance was read at 450 nm and results were calculated using a standard curve according to the manufacturer's instructions.

### 2.12 NF‐κB (p65) DNA‐binding activity assay

DNA‐binding activity of NF‐κB (p65) was detected by an NF‐κB (p65) transcription factor assay kit (Cayman Chemical Co., Ann Arbor, MI, USA) in the renal nuclear extracts according to the manufacturer's protocol. Briefly, nuclear extract (10 μg) was added to the ELISA plate coated with a specific double‐stranded DNA sequence containing the NF‐κB response element. NF‐κB (p65) protein:DNA complex was detected by addition of specific primary antibody directed against NF‐κB followed by HRP‐conjugated secondary antibody and the absorbance was read at 450 nm. The results were calculated using a standard curve following the manufacturer's instructions.

### 2.13 MicroRNA quantification

The mirVana™ miRNA isolation kit (ThermoFisher Scientific, Waltham, MA) was used to isolate small RNAs from renal tissues. Using the miRCURY LNA RT Kit and miRCURY LNA SYBR Green PCR Kit (Qiagen), isolated RNA (10 ng) was reverse transcribed and expression level of mature mmu‐miR‐133a‐5p (YP00205320‐miRCURY LNA miRNA PCR Assay) was determined using the Mx3000P real‐time PCR system and data were analyzed with MxPro software (Stratagene, La Jolla, CA). The relative expression of miR‐133a was calculated and normalized to the small nuclear RNA U6 snRNA (YP00203907‐miRCURY LNA miRNA PCR Assay) using the CT method as described above. Relative expression intensity values were calculated as 2^−ΔCT^, in which ΔCT are CT values normalized to the reference control RNA U6. Male and female samples were run in the same PCR cycles.

### 2.14 Plasma cGMP assay

Frozen plasma samples were used to analyze cGMP levels using direct cGMP immunoassay kit from Enzo LifeSciences (Farmingdale, NY) according to the manufacturer's protocol as previously reported.^42^ The results are expressed as picomoles of cGMP/mL of plasma using the standard curve generated by the manufacturer's protocol according to our published method.^43^ Male and female samples were analyzed on the same ELISA plate.

### 2.15 Plasma membrane preparation and GC activity assay

Plasma membranes were prepared from kidney tissues as previously described.^41^, ^42^ Briefly, tissues were homogenized in 5 volumes of 10 mM sodium phosphate buffer (pH 7.4) containing 250 mM sucrose, 150 mM NaCl, 1 mM PMSF, 5 mM benzamidine, 5 mM EDTA, and 10 μg/mL each of leupeptin and aprotinin, and centrifuged at 400 ***g*** for 10 min at 4°C. The supernatant was collected and recentrifuged at 80 000 ***g*** for 1 h at 4°C. The resultant supernatant was discarded, and the pellet was resuspended in 1 mL of 50 mM HEPES buffer (pH 7.4) containing 150 mM NaCl, 1 mM PMSF, 5 mM benzamidine, 5 mM EDTA, and 10 μg/mL each of leupeptin and aprotinin and centrifuged at 80 000 ***g*** for 1 h at 4°C. The final pellet was suspended in 200 μL of HEPES buffer (pH 7.4). GC activity was assayed as previously described^4^ with modifications.^42^ Briefly, a 50 μg aliquot of plasma membrane was added to 100 μL of GC assay buffer containing 50 mM Tris‐Cl buffer (pH 7.6), 4 mM MnCl_2_, 2 mM 3‐isobutyl‐1‐methylxanthine, 1 mM BSA, 5 units creatinine phosphokinase, 7.5 mM creatine phosphate, and 0.5 mM GTP. The samples were incubated in a water bath at 37°C for the indicated time, and reaction was stopped by adding 900 μL of 55 mM sodium acetate (pH 6.2), placing the sample tubes in a boiling water bath for 5 min, then keeping on ice for 15 min. Samples were centrifuged at 13 000 ***g*** for 5 min, supernatant was collected, and the generated cGMP was determined using a direct cGMP immunoassay kit from Enzo (LifeSciences) according to the manufacturer's protocol. The results are expressed as picomoles of cGMP/mg protein/30 min.

### 2.16 Measurement of albumin and creatinine in urine and plasma

Albumin levels in urine were measured in 24 h samples collected from animals kept in metabolic cages, using enzyme‐linked immunosorbent assay (ELISA) kit (Bethyl Laboratories, Montgomery, TX). Plasma albumin levels were quantitated utilizing QuantiChrom BCG albumin assay kit. Plasma and urine creatinine concentrations were analyzed using creatinine assay kit from Bioassay Systems (Hayward, CA) following the manufacturer's protocol. Creatinine clearance (CCr) was calculated from the urinary creatinine concentration, plasma creatinine concentration, and urine volume and expressed as μl/min. Male and female samples were analyzed in the same assay plate.

### 2.17 Statistical analysis

Statistical analyses were done using GraphPad PRISM 9.0 software (GraphPad Software, San Diego, CA). For multiple group comparison (vehicle‐treated, 2‐copy with 1‐copy or 3‐copy mice), one‐way analysis of variance (anova) was used, followed by the post‐hoc Tukey multiple comparison analysis. Two‐way anova was used for MAP analysis with comparisons between multiple groups when there were 2 experimental factors. MGCD‐treated groups were compared with their corresponding vehicle‐treated groups of the same genotype using 2‐tailed Student's unpaired *t* test, corrected using Welch's analysis. Similarly, for comparisons between sexes, male mice were compared with female mice of the same genotype using 2‐tailed Student's unpaired *t* test, corrected using Welch's analysis. The data are expressed as mean ± SE and *p* value of .05 was considered statistically significant.

## 3 RESULTS

### 3.1 MGCD treatment induced the renal expression of *Npr1* and GC activity

**FIGURE 1:**
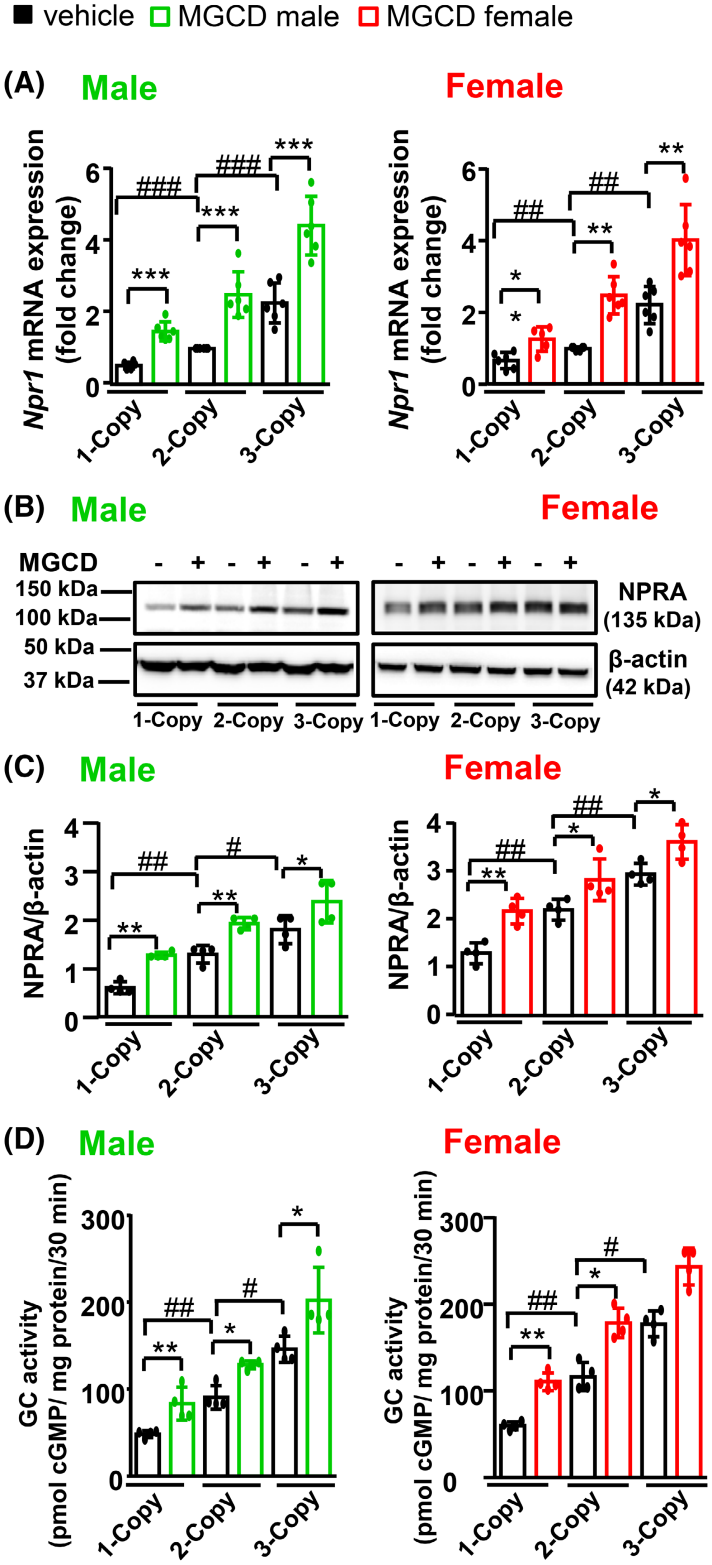
Renal *Npr1* mRNA, NPRA protein levels, and GC activity in MGCD‐treated gene‐targeted mice. (A) mRNA expression of renal *Npr1* in drug‐treated and control mice as determined by real‐time RT‐PCR, normalized to β‐actin mRNA (*n* = 6). (B, C) Western blot and densitometry analyses of NPRA protein levels in the cytosolic extract prepared from renal tissues, normalized to β‐actin protein in drug‐ or vehicle‐treated mice (*n* = 8). (D) ELISA of renal GC activity in drug‐ or vehicle‐treated mice (*n* = 8). *p < .05, **p < .01, ***p < .001 (vehicle‐ vs. drug‐treated, same genotype); ^#^ *p* < .05, ^##^ *p* < .01, ^###^ *p* < .001 (vehicle‐treated, 1‐copy or 3‐copy vs. 2‐copy).

Renal *Npr1* mRNA expression was reduced by 50% in male and female *Npr1* gene‐knockout heterozygous/haplotype (HT) 1‐copy (*Npr1* ^+/^) mice but greatly increased by 2‐fold in gene‐duplicated 3‐copy (*Npr1* ^++/+^) mice, compared with 2‐copy wild type (WT, *Npr1* ^+/+^) mice (Figure 1A). One‐copy mice exhibited markedly reduced NPRA protein levels and 3‐copy mice showed significant overexpression of NPRA than 2‐copy animals (Figure 1B,C). GC activity was also significantly lower in 1‐copy mice than 2‐copy and 3‐copy mice (Figure 1D). Treatment with MGCD markedly increased the expression of NPRA, GC activity, and plasma cGMP levels in 1‐copy mice (Table 1). Overall, female mice showed higher NPRA protein and GC activity than male mice (Figure 1C,D).

**TABLE 1:** Analyses of plasma cGMP, systolic blood pressure, and renal function among *Npr1* mice genotypes treated with MGCD and control mice.

Parameters/genotype	Male		Female	
	Vehicle	MGCD	Vehicle	MGCD
Plasma cGMP (picomoles/mL)				
1‐copy	10.2 ± 0.3^###^	19.8 ± 1.7**	11.9 ± 0.5^##^	21.4 ± 1.8**
2‐copy	19.2 ± 1.2	31.3 ± 1.6*	21.2 ± 1.2	32.1 ± 3.4*
3‐copy	31.0 ± 2.0^##^	46.1 ± 2.9*	34.6 ± 2.4^##^	43.5 ± 3.5*
Systolic blood pressure (mmHg)				
1‐copy	133.8 ± 4.7^###^	112.6 ± 3.3^****^	123.2 ± 5.1^####^	107.4 ± 5.8***
2‐copy	101.5 ± 2.0	96.8 ± 2.9**	95.2 ± 2.7	91.6 ± 2.6*
3‐copy	93.5 ± 2.3^###^	89.5 ± 2.9*	89.2 ± 3.8^##^	84.2 ± 1.0*
Creatinine clearance (μL/min)				
1‐copy	95.7 ± 9.4^###^	153.7 ± 7.8**	113.5 ± 5.3^#^	179.4 ± 19.4*
2‐copy	216.9 ± 9.9	307.1 ± 23.8*	214.2 ± 27.3	289.2 ± 46.5
3‐copy	335.7 ± 21.3^#^	416.8 ± 57.9	346.0 ± 33.8^#^	414.4 ± 18.7
Urinary albumin/creatinine				
1‐copy	0.74 ± 0.03^#^	0.39 ± 0.04**	0.31 ± 0.01^##^	0.23 ± 0.02*
2‐copy	0.47 ± 0.05	0.31 ± 0.02*	0.20 ± 0.01	0.19 ± 0.01
3‐copy	0.25 ± 0.01^#^	0.18 ± 0.01*	0.17 ± 0.04	0.12 ± 0.01
Plasma albumin (g/dL)				
1‐copy	2.00 ± 0.11^#^	2.32 ± 0.10*	1.99 ± 0.04^#^	2.27 ± 0.06
2‐copy	2.43 ± 0.07	2.50 ± 0.16	2.46 ± 0.14	2.59 ± 0.20
3‐copy	2.72 ± 0.14	2.76 ± 0.14	2.56 ± 0.08	2.77 ± 0.12

*Note*: The plasma cGMP, systolic blood pressure, creatinine clearance, urinary albumin/creatinine ratio, and plasma albumin levels were determined as described in the Methods section. *p < .05, **p < .01 (vehicle‐ vs. drug‐treated, same genotype); ^#^ *p* < .05, ^##^ *p* < .01, ^###^ *p* < .001, ^####^ *p* < .0001 (vehicle‐treated, 1‐copy or 3‐copy vs. 2‐copy); *n* = 8.

### 3.2 MGCD‐lowered mean arterial pressure and renal dysfunction in *Npr1* mice

**FIGURE 2:**
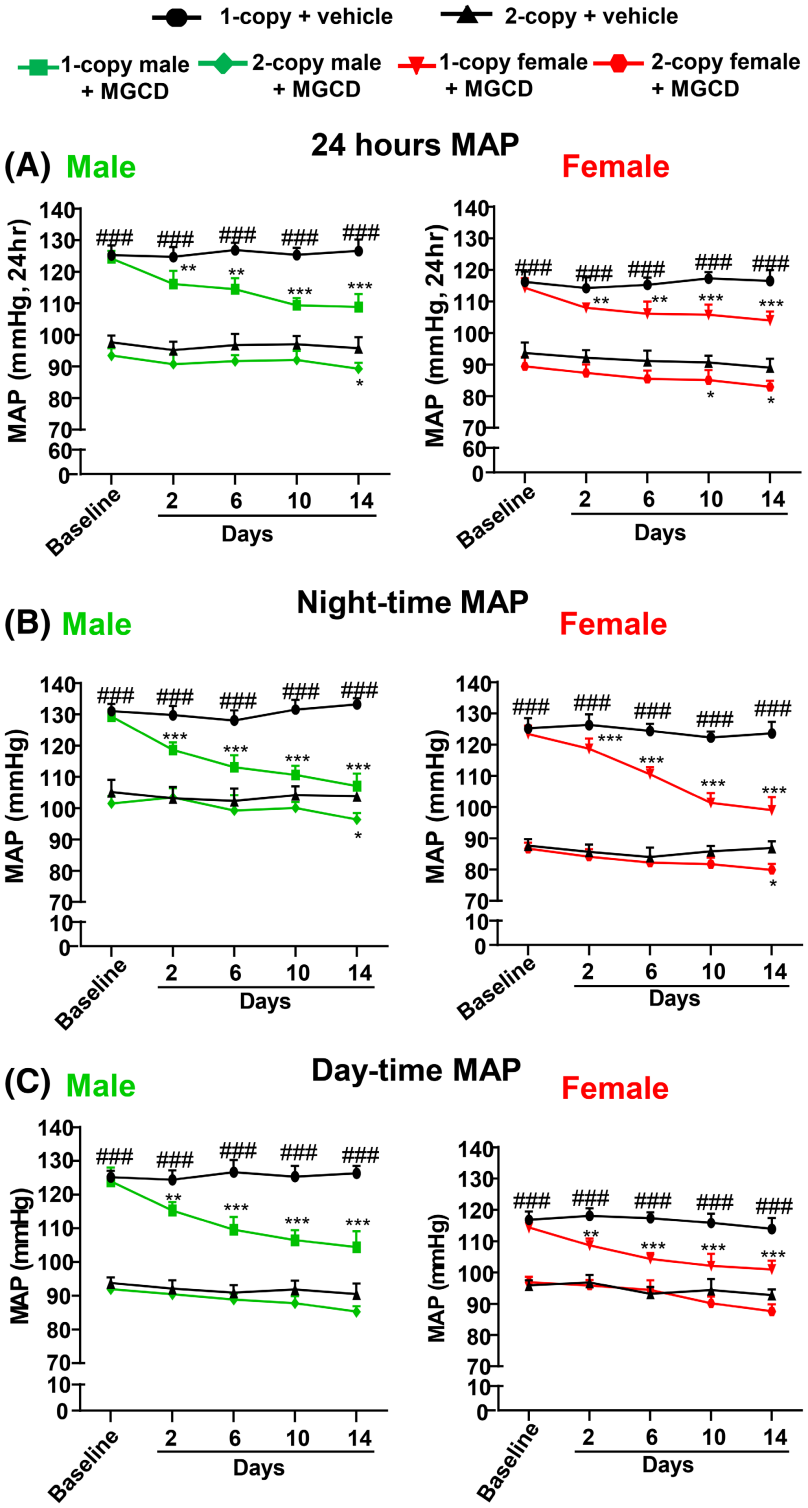
Effects of MGCD treatment on MAP in *Npr1* gene‐targeted mice. (A) BP was measured by radiotelemetry during 24 h periods, and changes in MAP in the vehicle‐treated and MGCD‐treated mice were recorded for 14 days (*n* = 10). (B) Changes in MAP during active (6:00 pm to 6:00 am) and (C) inactive (6:00 am to 6:00 pm) periods in control and MGCD‐treated male and female mice. *p < .05, **p < .01, ***p < .001, ^****^ *p* < .0001 (vehicle‐ vs. drug‐treated, same genotype); ^####^ *p* < .0001 (vehicle‐treated, 1‐copy vs. 2‐copy mice).

Mean arterial pressure (MAP) was significantly higher in 1‐copy male and female mice as compared to 2‐copy mice (1‐copy vs. 2‐copy males: 125 ± 3 vs. 98 ± 2 mmHg; and 1‐copy vs. 2‐copy females: 117 ± 3 vs. 93 ± 2 mmHg) (Figure 2A). Treatment with MGCD drastically reduced MAP in both 1‐copy male and female mice. The changes in MAP occurred in both sexes during active (nighttime, 6 pm to 6 am) and inactive (daytime, 6 am to 6 pm) periods (Figure 2B,C). Systolic blood pressure (SBP) measured by non‐invasive tail‐cuff method was also higher in 1‐copy mice compared with WT 2‐copy mice. MGCD effectively reduced delta SBP (∆SBP) in 1‐copy male (∆‐21 ± 2.0 mmHg) and female (∆‐15 ± 2.1 mmHg) mice with greater efficacy in male animals (Table 1). Creatinine clearance (CCr) was significantly reduced in 1‐copy mice compared with 2‐copy mice (males: 52%, *p* < .001 and females: 40%, *p* < .05); however, MGCD markedly increased CCr in 1‐copy mice (Table 1). An increased level of urinary albumin to creatinine ratio (ACR) was noted in 1‐copy male and female mice (*p* < .05) compared to 2‐copy mice and MGCD treatment effectively reversed the ACR in 1‐copy animals (Table 1).

### 3.3 MGCD attenuated the renal histopathological and morphometric damage in *Npr1* mice

**FIGURE 3:**
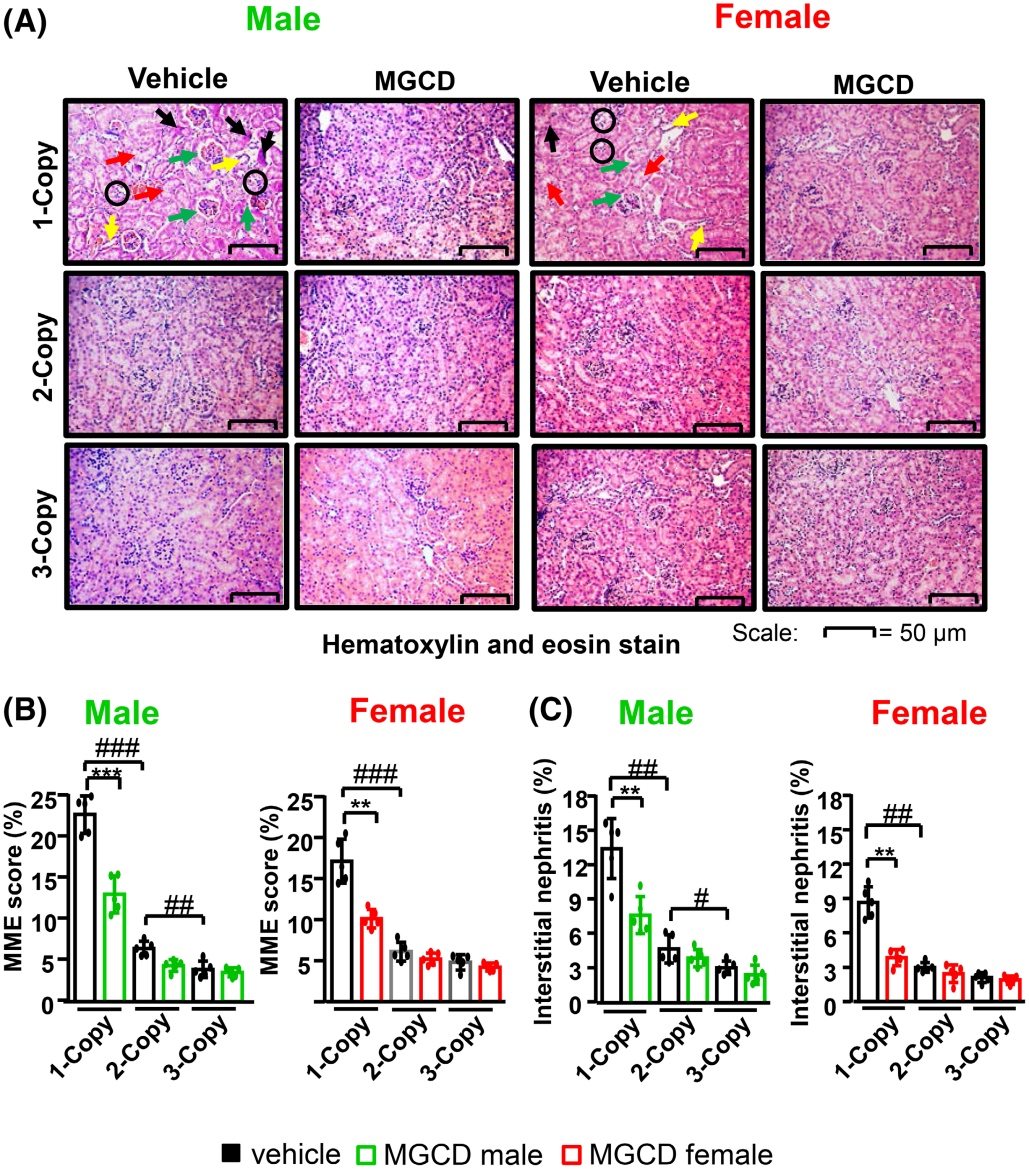
Histopathological investigation of renal tissue sections of MGCD‐treated *Npr1* gene‐targeted mice. (A) Representative images of paraffin‐embedded renal tissue sections (5 μm) of male and female mice stained with hematoxylin and eosin stain. One‐copy mice show vacuolar degeneration in multiple tubules (red arrow), pyknotic nuclei (black arrow) and tubular dilation (yellow arrow), glomerular injury like mesangial matrix expansion (MME; green arrow) and interstitial nephritis (circled). (B, C) quantitative analysis of MME and interstitial nephritis in mice relative to the total kidney area as determined by calculations made in 20 randomly selected microscopic fields in five sections per kidney using ImagePro Plus image analysis software. *p < .05, **p < .01, ***p < .001 (vehicle‐ vs. drug‐treated, same genotype); ^#^ *p* < .05, ^##^ *p* < .01, ^###^ *p* < .001 (vehicle‐treated, 1‐copy or 3‐copy vs. 2‐copy); *n* = 5; Scale bar = 50 μm; MME, mesangial matrix expansion.

Hematoxylin and eosin (H&E) staining of kidney sections showed more severe pathology and morphometric injury and damages in 1‐copy male mice and female mice, compared with 2‐copy mice (Figure 3A). Histopathological disorders included vacuolar degeneration in multiple tubules containing prominent cytoplasmic vacuoles, pyknotic nuclei, tubular dilation, mesangial matrix expansion (MME), and interstitial nephritis in 1‐copy mice, but these abnormalities were less severe in female mice as compared to 2‐copy control animals. MGCD treatment reduced the MME scores in both 1‐copy male and female mice (Figure 3B). Interstitial nephritis was also attenuated in MGCD‐treated 1‐copy male and female mice compared with vehicle‐treated control groups (Figure 3C). The histological damage was not discernible in 2‐copy and 3‐copy mice.

### 3.4 MGCD enhanced histone acetylation and inhibited HDAC expression

**FIGURE 4:**
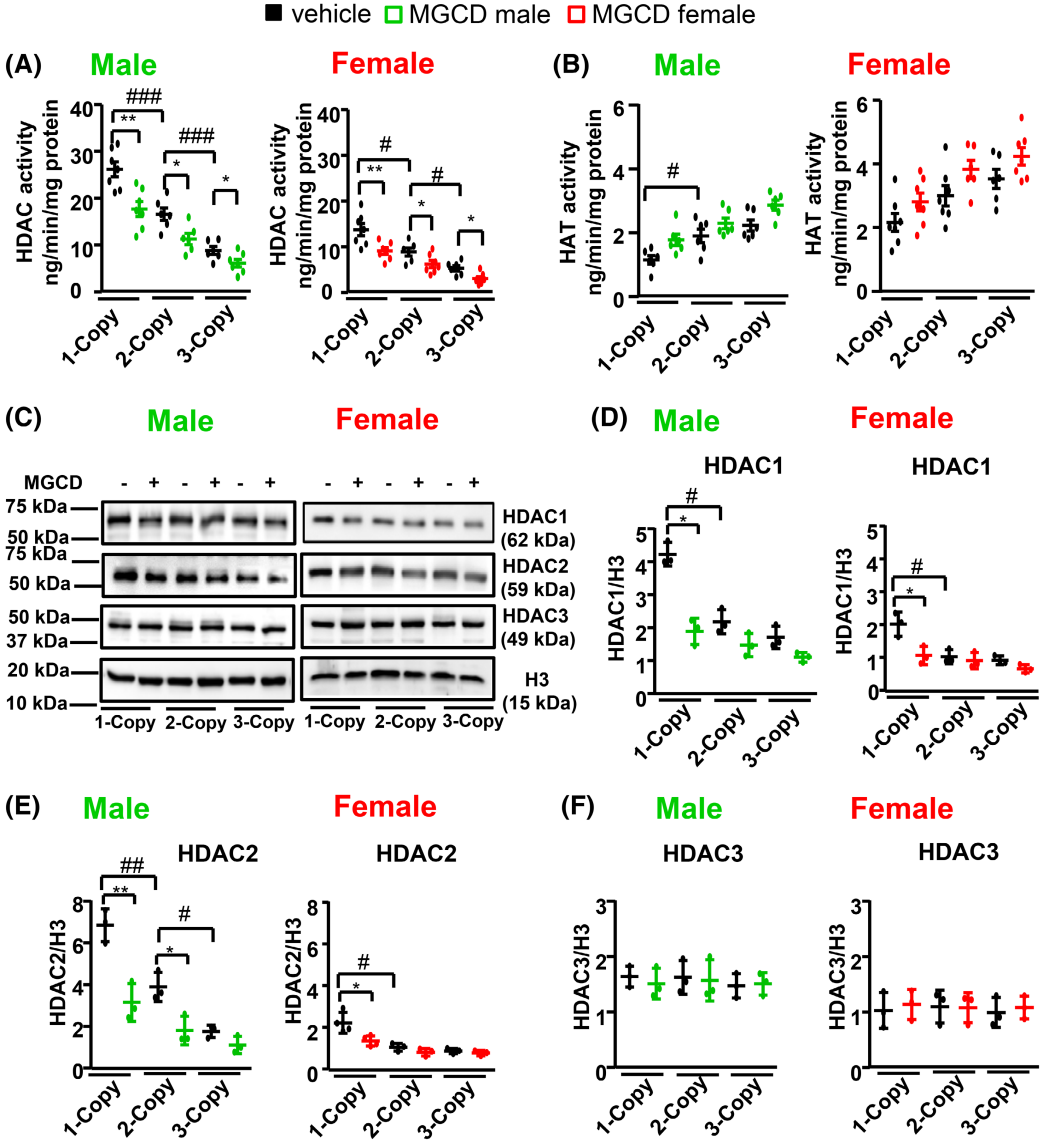
Modulation of renal HDAC and HAT activities and class I HDAC protein levels by MGCD treatment in *Npr1* gene‐targeted mice. (A, B) Quantification of HDAC and HAT activity in nuclear extracts of drug‐ or vehicle‐treated mice kidneys (*n* = 6). (C–F) Western blots and densitometry analyses of class I HDACs, (HDAC1, HDAC2 and HDAC3) protein levels in renal nuclear extracts of drug‐ or vehicle‐treated *Npr1* gene‐targeted mice (*n* = 6). *p < .05, **p < .01 (vehicle‐ vs. drug‐treated, same genotype); ^#^ *p* < .05, ^##^ *p* < .01, ^###^ *p* < .001 (vehicle‐treated, 1‐copy or 3‐copy vs. 2‐copy).

**FIGURE 5:**
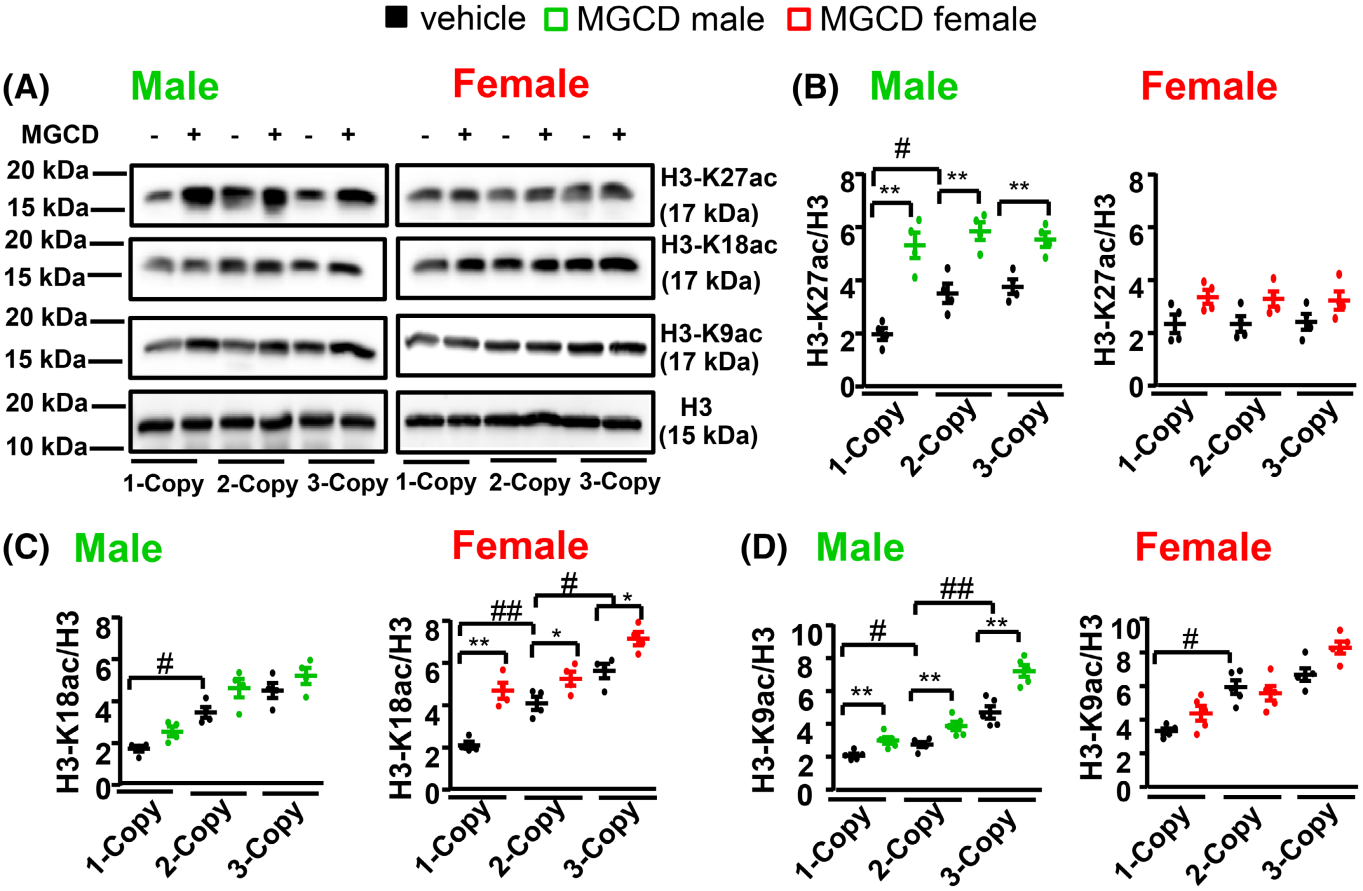
Analyses of H3 acetylation marks in kidneys of MGCD‐treated *Npr1* gene‐targeted mice. (A–D) Western blots and densitometry analyses of acetylated H3 proteins, H3‐K27ac, H3‐K18ac, and H3‐K9ac in total histones purified from renal tissues from drug‐ or vehicle‐treated mice and normalized with H3 (*n* = 6). *p < .05, **p < .01 (vehicle‐ vs. drug‐treated, same genotype); ^#^ *p* < .05, ^##^ *p* < .01 (vehicle‐treated, 1‐copy or 3‐copy vs. 2‐copy).

**FIGURE 6:**
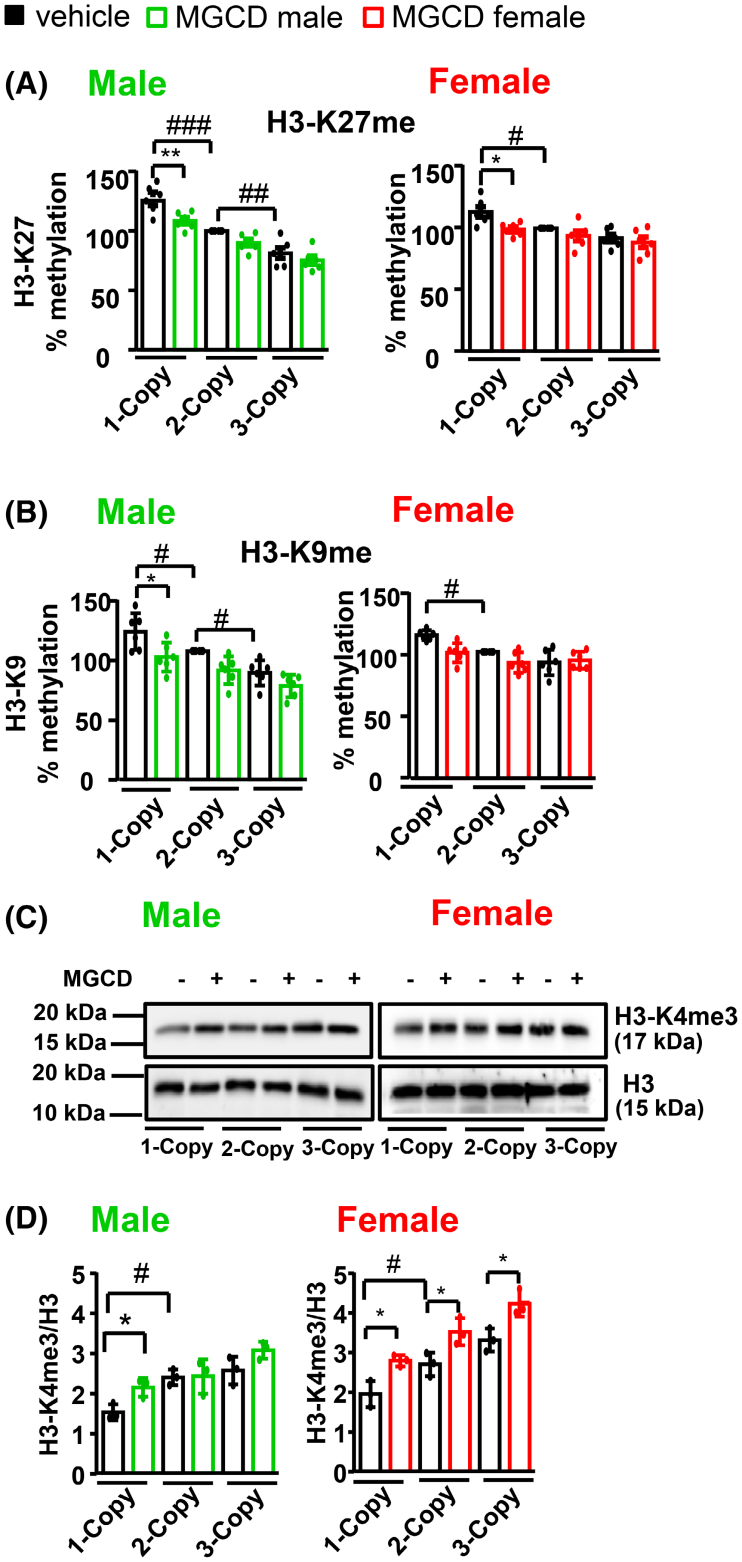
Analyses of H3 methylation marks in kidneys of MGCD‐treated *Npr1* gene‐targeted mice. (A, B) Global H3 methylation levels of specific lysine at H3‐K27me and H3‐K9me in nuclear extracts of renal tissues from drug‐ or vehicle‐treated mice (*n* = 6). (C, D) Western blot and densitometry analyses of active histone mark H3‐K4me3 protein normalized with H3 levels in total histones extracted from kidney of drug‐ or vehicle‐treated mice (*n* = 6). *p < .05, **p < .01 (vehicle‐ vs. drug‐treated, same genotype); ^#^ *p* < .05, ^##^ *p* < .01, ^###^ *p* < .001 (vehicle‐treated, 1‐copy or 3‐copy vs. 2‐copy).

Renal HDAC activity was significantly upregulated in 1‐copy male and female mice and MGCD distinctly decreased HDAC activity in these animals (Figure 4A). On the contrary, renal HAT activity was lower in both female and male 1‐copy mice; however, MGCD treatment greatly increased HAT activity in mutant animals (Figure 4B). Renal HDAC1 and 2 protein levels in 1‐copy males and females were significantly higher than 2‐copy and 3‐copy mice and MGCD treatment effectively reduced the renal HDAC1 and 2 protein levels (Figure 4C–E). On the other hand, HDAC3 protein was not associated with either genotype or sex of these animals (Figure 4F). A decreased acetylation of H3‐K27ac, H3‐K18ac, and H3‐K9ac occurred in 1‐copy mice but with greater magnitudes in males than females; however, MGCD increased the acetylation histone marks (Figure 5A–D). The methylated repressive histone marks (H3‐K27me and H3‐K9me) were also significantly increased in 1‐copy mice than 2‐copy mice and MGCD significantly reduced these methylations in 1‐copy male mice with weaker effect in female mice (Figure 6A,B). Active methylated histone mark, H3‐K4 trimethylation (H3‐K4me3) showed attenuated protein levels in 1‐copy male and female mice compared to 2‐copy and 3‐copy mice (Figure 6C,D).

### 3.5 MGCD inhibited the nuclear translocation and DNA binding activity of NF‐κB

**FIGURE 7:**
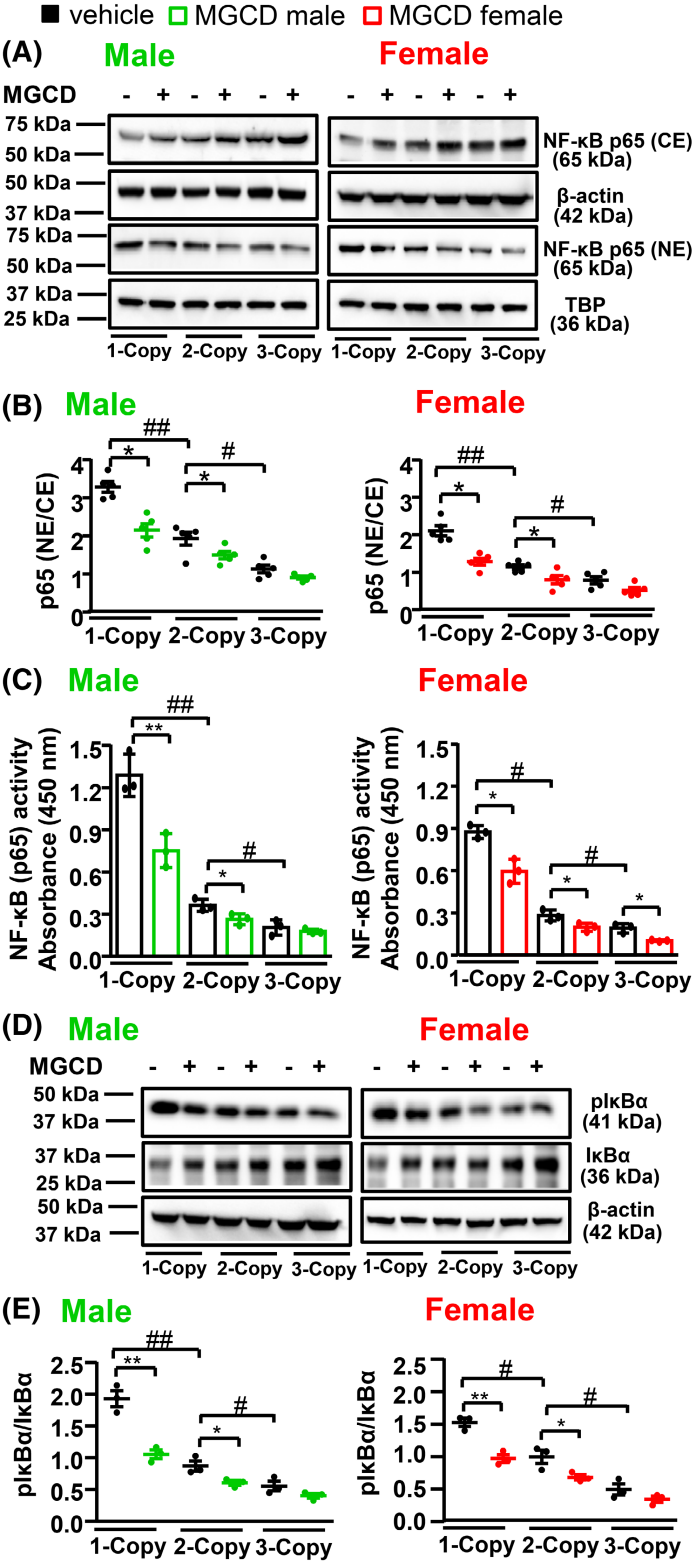
Renal NF‐κB (p65) cellular localization and DNA‐binding activity and phosphorylated and total IκBα protein levels in MGCD‐treated *Npr1* gene‐targeted mice. (A) Western blot showing renal cytoplasmic and nuclear localization of NF‐κB (p65) protein. β‐actin was used as loading control of cytoplasmic protein and TATA binding protein (TBP) was used as loading control for nuclear proteins (*n* = 6). (B) Densitometric analysis showing ratio of nuclear versus cytoplasmic localization of NF‐κB (p65) protein. (C) NF‐κB (p65) DNA binding activity in renal nuclear extracts from MGCD‐ or vehicle‐treated mice (*n* = 6). (D) Western blot analysis of phosphorylated IκBα and total IκBα protein levels in the renal cytoplasmic extract. β‐actin was used as loading control (*n* = 6). (E) Densitometric analysis showing ratio of phosphorylated IκBα and total IκBα proteins (*n* = 6). CE, cytoplasmic extract; NE, nuclear extract. *p < .05, **p < .01 (vehicle‐ vs. drug‐treated, same genotype); ^#^ *p* < .05, ^##^ *p* < .01 (vehicle‐treated, 1‐copy or 3‐copy vs. 2‐copy).

The renal cytoplasmic NF‐κB (p65) protein level was significantly lower in 1‐copy male and female mice. MGCD markedly increased the cytoplasmic levels of NF‐κB (p65) in 1‐copy male and female mice (Figure 7A). The ratio of NF‐κB (p65) in renal nuclear extract (NE) to cytoplasmic extract (CE) was higher in 1‐copy mice than 2‐copy mice (Figure 7B). Similarly, DNA binding activity of nuclear NF‐κB (p65) was stronger in 1‐copy males and females, while 2‐copy and 3‐copy mice showed weaker activity (Figure 7C). MGCD treatment significantly reduced the DNA binding activity of NF‐κB (p65) in 1‐copy animals. Phosphorylated IκBα (pIκBα) levels were greater than total IκBα in 1‐copy mice compared with 2‐copy mice (Figure 7D). The MGCD significantly reduced the pIκBα/IκBα ratio in 1‐copy mice than vehicle‐treated control animals (Figure 7E).

### 3.6 MGCD enhanced the expression of antifibrotic microRNA‐133a and reduced the fibrotic markers α‐SMA and COL1α‐2

**FIGURE 8:**
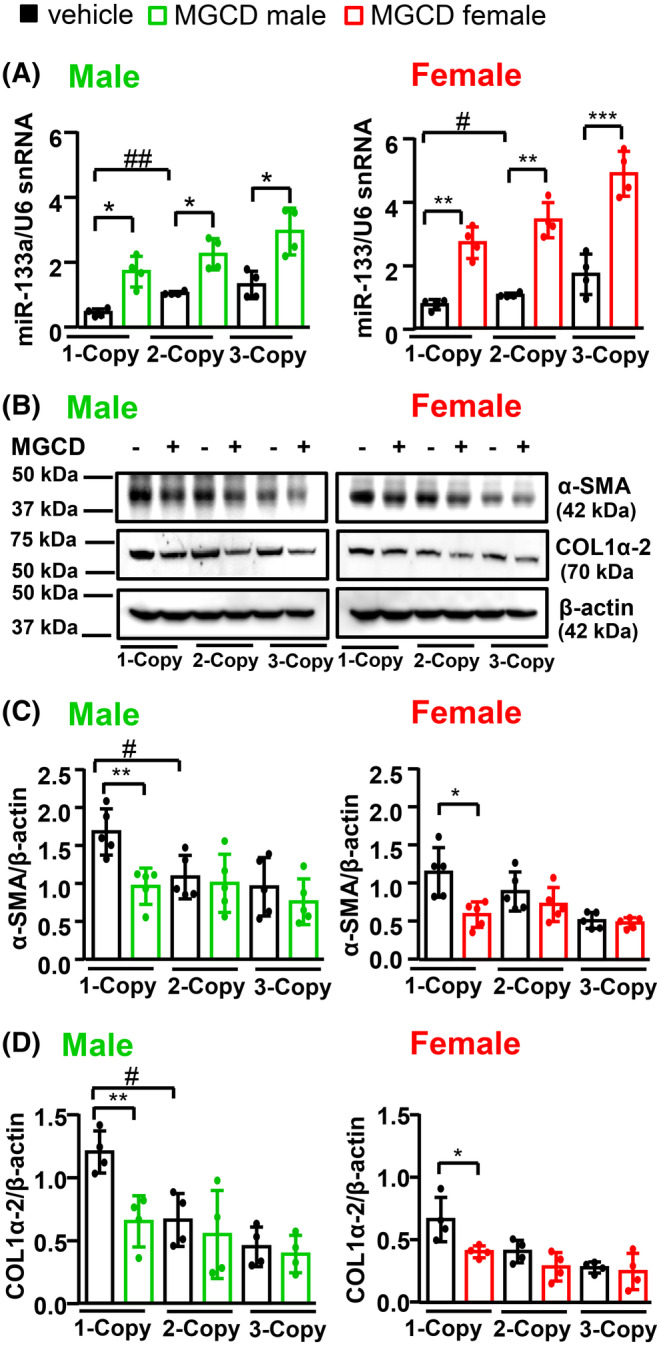
Renal expression of anti‐fibrotic miR‐133a and its downstream targets α‐SMA and COL1α‐2 in drug‐treated *Npr1* gene‐targeted mice. (A) RT‐qPCR analysis of miR‐133a expression levels in miRNAs isolated from renal tissues as normalized to U6 snRNA (*n* = 6). (B–D) Western blot and densitometric analyses of α‐SMA and COL1α‐2 in the cytoplasmic extract prepared from renal tissues of drug‐treated and vehicle‐treated mice. β‐Actin was used as loading control (*n* = 6). *p < .05, **p < .01, ***p < .001 (vehicle‐ vs. drug‐treated, same genotype); ^#^ *p* < .05, ^##^ *p* < .01 (vehicle‐treated, 1‐copy or 3‐copy vs. 2‐copy).

**FIGURE 9:**
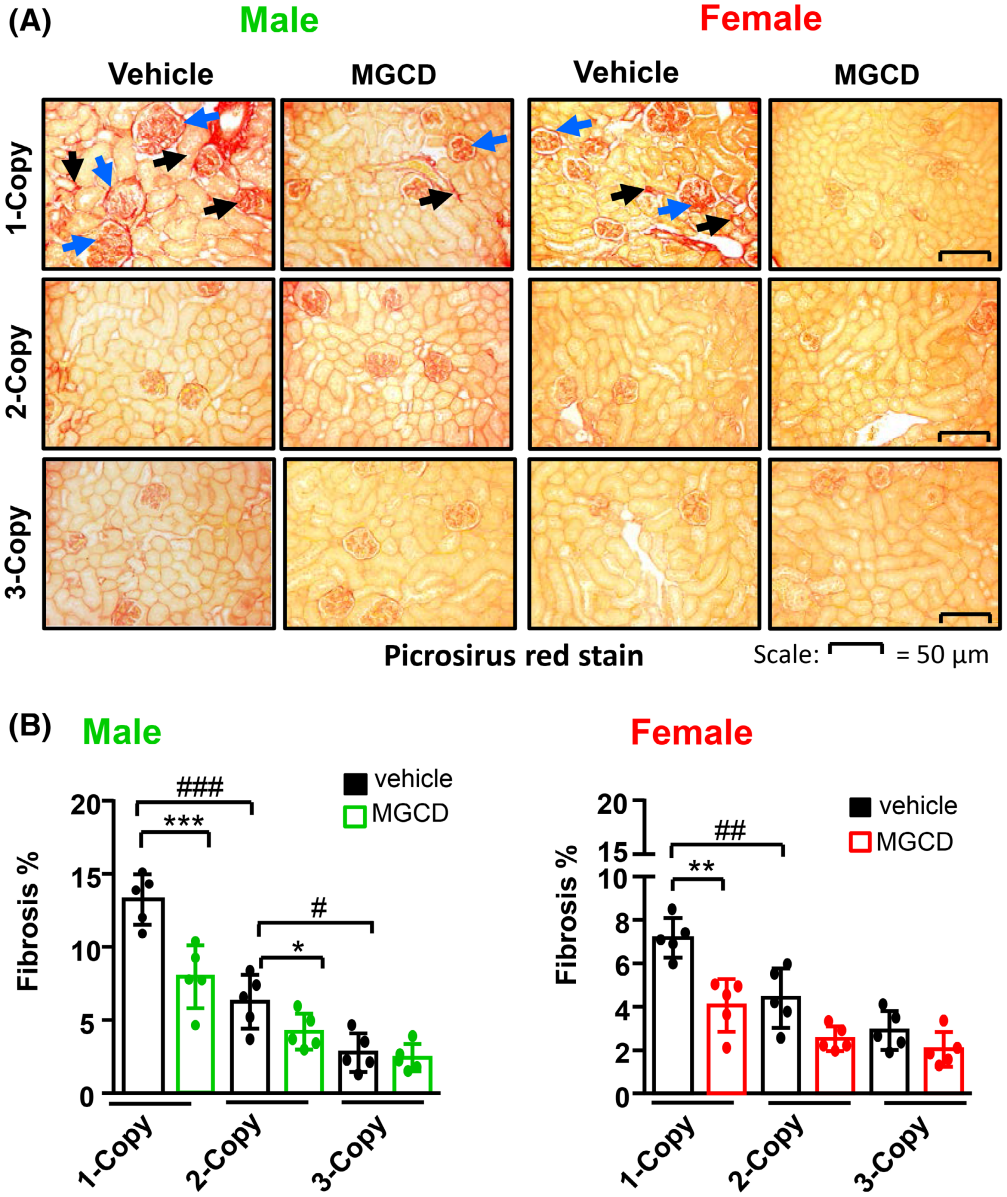
Renal histopathology of interstitial and glomerular fibrosis in drug‐ and vehicle‐treated *Npr1* gene‐targeted mice. (A) Picrosirius red staining of renal tissue sections of *Npr1* 1‐copy mice revealed extra collagen deposition in the interstitium (black color, arrows) and in the intraglomerular and periglomerular regions (blue color, arrows). (B) Quantitative analysis of renal fibrosis in mice relative to the total kidney area as determined by calculations made in 20 randomly selected microscopic fields in three sections per kidney using ImagePro Plus image analysis software. *p < .05, **p < .01, ***p < .001 (vehicle‐ vs. drug‐treated, same genotype); ^#^ *p* < .05, ^##^ *p* < .01, ^###^ *p* < .001 (vehicle‐treated, 1‐copy or 3‐copy vs. 2‐copy); *n* = 6. Scale bar = 50 μm.

The expression of miR‐133a was significantly lower in 1‐copy mice compared with 2‐copy mice (Figure 8A). MGCD markedly enhanced the expression of miR‐133a by 2.8‐fold in males and by 4‐fold in females. Analyses of miR‐133a target proteins exhibited significantly higher levels of α‐SMA and COL1α‐2 in 1‐copy mice than 2‐copy mice (Figure 8B–D). MGCD significantly reduced profibrotic markers, α‐SMA, and COL1α‐2 levels in 1‐copy mice compared with untreated controls. Similarly, picrosirius red staining of kidney sections showed that MGCD significantly attenuated fibrosis by 46% in males and 35% in females, respectively, compared with vehicle‐treated 1‐copy animals (Figure 9A,B).

## 4 DISCUSSION

The results demonstrate that MGCD lowered high BP and kidney injury and dysfunction in 1‐copy *Npr1* ^*+/−*^ mutant mice in a sex‐dependent manner. The data showed a significantly lower baseline MAP in female mice than male mice. MGCD exhibited greater efficacy in male mice compared to female mice, suggesting that decreased NPRA/cGMP signaling is associated with sex‐dependent renal dysfunction, histomorphological damage, and hypertension to a greater extent in males than females at molecular level. MGCD inhibited the profibrotic class I HDAC1 and 2, enhanced renal *Npr1* expression and GC activity, and increased the plasma cGMP levels in both sexes. Previously, MGCD has been shown to reduce pulmonary arterial pressure and pulse pressure in a hypoxia‐induced pulmonary hypertension in the experimental animal models.^16^ We noted that sex differences in MAP and SBP, urinary protein and albumin excretion, and histopathology were considerably higher with disease magnitudes in males than females. MGCD treatment completely reversed proteinuria in 1‐copy male mice but had much lower efficacy in females. Previous studies have indicated that adult male mice showed higher urinary albumin and protein than female mice.^26^, ^44^ Our current findings demonstrate that the beneficial effects of MGCD are seen in reversing the renal pathology and high BP in 1‐copy male and female mice by activating the acetylated positive histone marks and inhibiting the methylated negative histone marks. These changes lead to greater physiological benefits, such as reduction of SBP/MAP, ameliorating renal dysfunction, and reduction in renal fibrotic markers in 1‐copy male mice, indicating that the drug has higher efficacy in male than female mice. Our previous studies and works from others have shown that systemic targeted disruption of the *Npr1* provokes hypertension and cardiac dysfunction in null mutant (0‐copy) and haplotype (1‐copy) mice.^9^, ^10^, ^45^, ^46^ The earlier reports from our laboratory have also demonstrated that the hematocrit status was not changed in either 2‐copy WT or 0‐copy mutant mice.^9^ However, after volume expansion, the hematocrits in the recipient 2‐copy and 4‐copy (gene‐duplicated) mice were significantly higher compared with recipient 0‐copy mice.^9^ Additionally, we have also shown that 0‐copy and 1‐copy mice had significantly increased heart weight‐body weight (HW/BW) ratio, high BP, hypertrophic markers, including beta‐myosin heavy chain and protooncogenes (c‐fos and c‐jun), proinflammatory mediator, NF‐κB, and matrixmetalloproteinases‐2 and ‐9 (MMP‐2, MMP‐9) compared with 2‐copy and 4‐copy mice.^45^, ^47^ However, the potential effect of HDACi on these metrics is not yet well known in *Npr1* mutant mice.

The present findings show that 1‐copy male mice exhibited higher levels of HDAC1 and 2 proteins than female mice, leading to more severe kidney injury and histopathology. Previous findings have suggested that protein levels of HDACs 1, 2, and 5 were also elevated in male but not female mice after arsenic exposure, leading to sex‐dependent differences in cognitive flexibility.^48^ We noted that MGCD treatment reduced HDAC1 and 2 protein levels in both sexes. Several classes of HDACi, including trichostatin (TSA), vorinostat, hydroxamic acid, and sodium butyrate (NaBu), decrease the expression as well as activity of HDACs and shift the overall balance in the favor of histone acetylation.^49^, ^50^, ^51^ Earlier studies have informed that HDACi induces protein degradation by regulation of enzymes in the ubiquitin‐proteasome pathway. For example, valproic acid has been shown to reduce HDAC2 protein levels via proteasomal degradative pathway.^52^, ^53^ Our results support the notion that increased renal HDAC1 and 2 expression and decreased histone acetylation are associated with high BP and acute renal injury with fibrosis.^21^, ^54^, ^55^ The acetylation of H3‐K9 and H3‐K27 was significantly decreased in 1‐copy male mice (due to higher HDAC1 and 2 expression levels) than female mice, which was effectively reversed by MGCD treatment. Acetylation of lysine residues on the H3‐K9 seem to be beneficial with posttranslational modifications in renal tubulointerstitial cells.^56^ On the other hand, global histone methylation has been associated with kidney injury and progressive glomerulosclerosis.^57^ Increase in negative histone codes (H3‐K9me3 and H3‐K27me3) in fibrotic mouse kidney and H3‐K27me3 in humans with chronic kidney disease (CKD) has also been reported.^56^, ^58^ Upregulation of H3K27me levels downregulates *Kl* (klotho) gene, which seems to be associated with hypertension.^59^ Our results demonstrate an enhanced methylation of negative histone marks (H3‐K9 and H3‐K27) in 1‐copy mice; however, MGCD treatment enhanced the histone acetylation, thereby lowering histone methylation at these lysine residues.

Histone deacetylase inhibitors prevent NF‐κB activation by suppressing proteasome activity, which stabilizes IκBα.^60^ The present results show that the enhanced phosphorylation of IκBα increased the nuclear translocation of NF‐κB (p65) in 1‐copy mice compared with 2‐ and 3‐copy mice, which was markedly reversed by MGCD treatment. Previous findings have shown that HDACi, including vorinostat and sodium butyrate, also inhibited the NF‐κB pathway by suppressing the degradation of IκBα.^61^, ^62^ We observed the sex‐specific differences in the cytoplasmic and nuclear levels of NF‐κB (p65) in male mice (lower cytoplasmic and higher nuclear levels) than female mice, which was also reflected in the DNA binding activity of nuclear NF‐κB. It has been shown that in other tissues, including heart and brain, female mice exhibited weaker activation of NF‐κB signaling than males.^63^, ^64^

**FIGURE 10:**
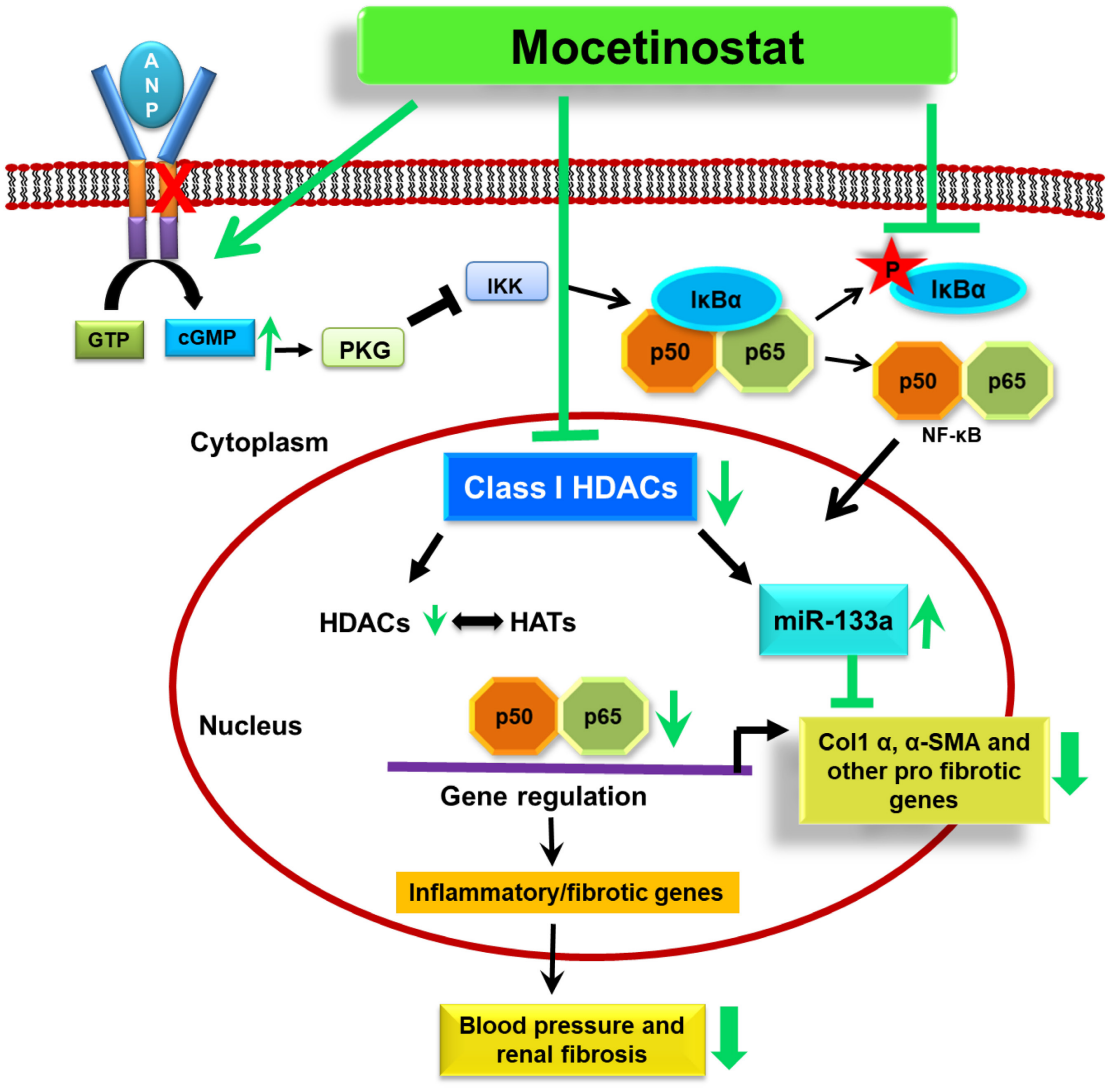
The proposed model depicts MGCD signaling in kidneys of *Npr1* haplotype 1‐copy mice. Disruption of *Npr1* is associated with enhanced HDAC activity and HDAC1 and 2 protein expression, and downregulation of anti‐fibrotic miR‐133a expression, which promotes renal fibrosis and dysfunction in 1‐copy mice. Mice treated with MGCD exhibit increased NPRA level and cGMP signaling, lower HDAC activity, and stabilization of IκBα molecule, which leads to formation of inactive NF‐κB (p65) complex in the cytoplasm. MGCD also enhances miR‐133a expression, leading to downregulation of profibrotic genes and an improved renal function.

MiRs have been implicated as a novel therapeutic target for the treatment of high BP, kidney injury, and fibrogenesis.^19^, ^65^, ^66^ Our results showed that miR‐133a expression was significantly attenuated in 1‐copy male and female mice compared with 2‐copy mice, and MGCD treatment markedly upregulated miR‐133a expression in both sexes, with relatively higher responses in females. HDAC inhibition has also been shown to regulate miR levels in skeletal muscle cells and cardiomyocytes.^22^, ^67^ Correlation analysis in patients undergoing renal sympathetic denervation showed a significant relationship between SBP reduction and increased miR‐133a expression after 6‐month follow‐up.^24^ Profibrotic genes are post‐transcriptionally silenced by miR‐133a, including *COL1α1*, *α‐SMA*, and connective tissue growth factor (*CTGF*) genes.^18^, ^68^ Interestingly, previous work has shown that miR‐133a regulates renin‐angiotensin system by repressing the target gene angiotensinogen (AGT) expression in renal cortex and influences the tumor necrosis factor‐alpha (TNF‐α)‐mediated changes in blood pressure in a salt‐sensitive manner.^69^ Further, miR‐133a is also reported to downregulate brain AGT^70^ and (pro)renin receptor in angiotensin II‐dependent hypertension.^71^ Our current results demonstrated an association between upregulated HDAC1/2 and attenuated miR‐133a expression with enhanced levels of α‐SMA and COL1α‐2 proteins in 1‐copy haplotype mice. MGCD treatment inhibited HDACs, enhanced miR‐133a, and decreased the levels of α‐SMA and COL1α‐2, leading to reduced renal fibrosis/remodeling and lowered BP in the mutant animals (Figure 10). The present results also demonstrate MGCD‐mediated sex‐dependent distinctive modulation of epigenetic changes, lowered MAP, repaired renal fibrosis and proteinuria, and inhibited inflammatory and fibrotic factors highlighting the importance of research in studying differential effects of drugs in amelioration of disease in males versus females. Our results support the fact that sex is a critical biological modulator of renal physiology and suggest a role for new epigenetic targets affecting hypertension and renal dysfunction in a sex‐specific manner, which may help to develop gender‐based targeted therapies in humans.

In conclusion, our results provide direct evidence that^1^ treatment with MGCD significantly enhanced *Npr1* expression and GC activity of NPRA to a greater degree in male compared to female mice^2^; MGCD attenuated MAP and SBP, decreased kidney histopathology, and significantly reduced the excretion of elevated urinary protein and albumin levels more effectively in 1‐copy HT male than female mice^3^; HDAC activity and HDAC1 and 2 protein levels exhibited sex specificity and *Npr1* gene‐dose‐dependent association, with male mice having higher expression than females^4^; HDAC inhibition effectively enhanced the expression of anti‐fibrotic miR‐133a, thereby reducing its downstream fibrotic targets, including α‐SMA and COL1α‐2 proteins; and^5^ the inhibition of HDAC prevented the nuclear translocation of NF‐κB (p65), which correlated with a reduction in pIκBα and stabilization of IκBα in 1‐copy mutant mice in sex‐specific manner.

## AUTHOR CONTRIBUTIONS

Prerna Kumar and Kailash N. Pandey conceived and designed the research. Prerna Kumar, Kandasamy Neelamegam, Chandramohan Ramasamy, Ramachandran Samivel, Huijing Xia, Daniel R. Kapusta, and Kailash N. Pandey performed the research and acquired the data. Prerna Kumar and Kailash N. Pandey analyzed and interpreted the data. Kailash N. Pandey provided resources. Prerna Kumar and Kailash N. Pandey wrote the original draft. Prerna Kumar, Daniel R. Kapusta, and Kailash N. Pandey were involved in reviewing and editing the manuscript.

## FUNDING INFORMATION

This research was supported by grants from the National Institutes of Health (HL062147 and DK133833).

## DISCLOSURES

The authors declare no conflicts of interest.

## Data Availability

The data that support the findings of this study are available on request from the corresponding author. The data are not publicly available due to privacy or ethical restrictions.
